# Putting the ‘I’ Back into Integrated Care

**DOI:** 10.5334/ijic.6744

**Published:** 2022-06-13

**Authors:** Charlotte Augst

**Affiliations:** 1 National Voices, the coalition for health and care charities, England, UK

**Keywords:** integration, Integrated Care Systems, person-centered care, care planning, community, coordinated care, care coordination

*(1) Charlotte Augst, CEO of National Voices, reflects on progress with integrated care since the charity co-produced the I statements with people with lived experience of health and social care services*.

(2) Talk of integration in health and care waxes and wanes over time. But right now, if integration was a crypto currency, we would be approaching the point at which a dangerous bubble is about to burst, with a lot of disappointed investors scratching their heads about how come they, too, believed every desirable outcome in health and care could be achieved through the magic of *integration*. It is not surprising such high hopes are attached to the power of integration: Lack of coordination, fragmented responsibilities and disrupted information flows come up consistently as a theme whenever you speak to people who use health and care services.

However, people with bigger research and evaluation budgets than me have demonstrated clearly that the impact of integration efforts to date is relatively modest. In fact, the commonly chosen metrics of levels of ‘unplanned admissions’ or ‘delayed discharges’ remain stubbornly high despite considerable financial and time investment in integrated care. And quite often, no-one bothered measuring whether things improved *for people*.

But that doesn’t stop the enthusiasts from pushing on with it: In England, for example the Integrated Care Pioneers were relieved from the crushing expectations piled on their efforts by the arrival of the Vanguards, who were in turn brushed aside by the announcement of Sustainability and Transformation Partnerships, who were then overtaken by the creation of Integrated Care Systems. None of these policy experiments was allowed to conclude before another one was announced. Most recently, the Bill worming its way through Parliament in England to put ICSs onto a statutory footing was overtaken by yet another declaration of intent to accelerate integration in the form of a White Paper policy proposal.

As I have argued elsewhere, this acceleration of initiatives and announcements in England smacks of desperation. If the hundreds and hundreds of smart health and care leaders who spent large chunks of their working week on these integrated care change programmes haven’t made the outcomes budge for people, then what are we doing?

I would argue that the fundamental problem is that we look at the wrong end of the telescope. Which always reminds me of an illustration which has been floating around the twitter sphere for some time, and I often use in presentations. This shows a baby in a cot with a mobile of cute animals. Seen from above it looks like system designers are creating interesting, new variations on the integration theme. Look, a tiger! Look, an elephant! Seen from below, all the baby (who the invention is supposedly for) can see is bums. What would it look like if baby entertainment was designed for the person lying in the cot?

Similarly, what real system change would follow if we moved on from an ornamental approach to health and care reform in which committees develop pretty powerpoints about the admirable principles and the supporting governance structures to turn these into reality.

The words that follow are over-used and under-appreciated at the same time: start with people and communities, put the person at the centre, personalise care. The National Voices and TLAP I statements of 10 years ago have never been bettered, in my view, to capture the actual outcomes we would care about if we truly wanted to address people’s experiences of using services. “I tell my story once”. “When I move between services, there is a plan in place for what happens next.” Staying close to the question ‘what needs to change for this service user at this time to make these outcomes possible?’ you would potentially, eventually get to some structural questions about money flows and accounting lines. But I doubt that this is where you would start. And you certainly would realise (as in make real) a whole lot of change and improvement that doesn’t require structural change at all: defining the outcomes the person wants to see, documenting that, noting who needs to do something to make this happen, sharing information with these care partners – all these changes are about behaviours, rather than boards.

Importantly, using the person’s hopes and stated choices as the principle for planning an ‘integrated’ (better: coordinated) service response also guards against another major risk I always see when the integration agenda is treated as a ‘system first’ challenge. This occurs when an organisation or partnership appears to successfully deliver its services but these do not provide what people actually need or indeed negatively affects their wellbeing. A metaphor which I use regarding this risk is the Catholic Church in Ireland in the 20^th^ century: It was a truly integrated faith-based system, wrapping holistic care around a whole population: education, health, care, spirituality, family support – all of it provided by a set of coordinated and coherent providers. Sounds great? Not if you take account of the numerous human rights violations and even more numerous acts of oppression and unkindness that this system generated in places such as Dublin.^1^ If you were a single mother in Ireland for much of the 20^th^ century, you got an integrated service alright, but it destroyed your ability to live the life you might have chosen for yourself.^2^ Integration without personalisation is useless at best, and dangerous at worst.

We will only achieve the outcomes we claim to pursue through our integration efforts, if we start by asking people and communities what it is that matters to them, and then build a shared, effective, person and community centred response to the priorities that emerge. We also need the courage and strength to challenge existing power imbalances, professional interests, and organisational boundaries. Coproduction first, board structures last.

## Footnotes

Department of Justice. Report by Commission of Investigation into Catholic Archdiocese of Dublin. Dublin: Department of Justice. 2009. Available from: https://www.justice.ie/en/JELR/Pages/PB09000504.

Department of Children, Equality, Disability, Integration and Youth. Final Report of the Commission of Investigation into Mother and Baby Homes. Dublin: Government of Ireland. 2021. Available from: https://www.gov.ie/en/publication/d4b3d-final-report-of-the-commission-of-investigation-into-mother-and-baby-homes/#.

## Author Information

Charlotte Augst is the Chief Executive of National Voices, the coalition for health and care charities in England. Through the work of its 190 members, National Voices is connected to the experiences and aspirations of millions of people- patients, families, carers and communities. The need for better care coordination comes up as a consistent theme across different cohorts, conditions and communities. National Voices, together with Think Local Act Personal, developed the original I-statements in 2013 that describe what person centred, integrated, coordinated care would look like from the perspective of people who need services.

